# Triangulating the provenance of African elephants using mitochondrial DNA

**DOI:** 10.1111/j.1752-4571.2012.00286.x

**Published:** 2012-08-01

**Authors:** Yasuko Ishida, Nicholas J Georgiadis, Tomoko Hondo, Alfred L Roca

**Affiliations:** 1 Department of Animal Sciences, University of Illinois at Urbana-ChampaignUrbana, IL, USA; 2 Port Townsend, WA, USA; 3 Institute for Genomic Biology, University of Illinois at Urbana-ChampaignUrbana, IL, USA

**Keywords:** conservation, forensics, forest elephants, ivory, microsatellite markers, phylogeography, savanna elephants

## Abstract

African elephant mitochondrial (mt) DNA follows a distinctive evolutionary trajectory. As females do not migrate between elephant herds, mtDNA exhibits low geographic dispersal. We therefore examined the effectiveness of mtDNA for assigning the provenance of African elephants (or their ivory). For 653 savanna and forest elephants from 22 localities in 13 countries, 4258 bp of mtDNA was sequenced. We detected eight mtDNA subclades, of which seven had regionally restricted distributions. Among 108 unique haplotypes identified, 72% were found at only one locality and 84% were country specific, while 44% of individuals carried a haplotype detected only at their sampling locality. We combined 316 bp of our control region sequences with those generated by previous trans-national surveys of African elephants. Among 101 unique control region haplotypes detected in African elephants across 81 locations in 22 countries, 62% were present in only a single country. Applying our mtDNA results to a previous microsatellite-based assignment study would improve estimates of the provenance of elephants in 115 of 122 mis-assigned cases. Nuclear partitioning followed species boundaries and not mtDNA subclade boundaries. For taxa such as elephants in which nuclear and mtDNA markers differ in phylogeography, combining the two markers can triangulate the origins of confiscated wildlife products.

## Introduction

Molecular methods have been used to establish the geographic origins of wildlife or wildlife products, by comparing their genotypes to those of voucher specimens of known geographic origins (Baker et al. 2000; Baker 2008; Luo et al. 2008; Caniglia et al. 2010; Ghobrial et al. 2010). Wasser and colleagues have pioneered the application of these methods to African elephants and their ivory (Wasser et al. 2004, 2007, 2008; Mailand and Wasser 2007). Although the ivory trade was banned in 1989 by the Convention on International Trade in Endangered Species (CITES), the illegal smuggling of ivory is a major threat to elephant populations (Species Survival Network 2006; Thomas 2011). A record number of 13 large-scale (>800 kg) ivory seizures were reported in 2011, representing 23 tons of ivory and the deaths of over 2500 elephants (Thomas 2011). As ivory is often seized in markets far from locations where elephants are poached, inferring the geographic origins of ivory would enable law enforcement to be targeted toward poaching ‘hot spots’. Wasser et al. have developed methods to extract and amplify DNA from small amounts of elephant ivory (Mailand and Wasser 2007). They also developed the use of nuclear microsatellite markers to infer the source populations of confiscated African elephant ivory, by comparing the genotypes of ivory to the genotypes of elephant samples of known provenance (Wasser et al. 2004, 2007, 2008).

While African elephant males disperse from their natal core social group or ‘herd’, mediating nuclear gene flow (Hollister-Smith et al. 2007), female elephants are matrilocal and remain with their natal herd (Archie et al. 2007; Hollister-Smith et al. 2007). As the mitochondrial genome is only transmitted maternally, and females do not typically migrate between herds, the mitochondrial genome is tied to the geographic range of the herd. Elephant mtDNA thus follows a very different evolutionary trajectory than nuclear DNA (Roca 2008). The demographic constraint on African elephant mtDNA renders it a poor genetic marker for inferring population structure and taxonomy, because mtDNA phylogeographic patterns are incongruent with and sometimes orthogonal to nuclear phylogeographic patterns (Roca et al. 2005, 2007; Lei et al. 2008, 2009; Ishida et al. 2011b). Yet precisely because nuclear and mtDNA phylogeographic patterns differ, we hypothesized that the ability of genetic methods to assign the provenance of African elephants (or their ivory) would be greatly improved by the inclusion of the distinctive information provided by mtDNA.

We here considered whether the phylogeographic information provided by mtDNA could be used to identify the origins, or improve the geographic assignment, of African elephants or their ivory. We sequenced and analyzed more than 4 kb of mtDNA sequences from 653 African elephants, including forest and savanna elephants, grouped into 22 locations from 13 countries. We also combined our control region sequences with those of other trans-national studies for a comprehensive phylogeographic analysis of the distribution of control region haplotypes of elephants from 81 localities across 22 countries in Africa. We found a high degree of support for the presence of eight distinctive subclades of mtDNA; determined the distribution of each subclade regionally across Africa; found that many mtDNA haplotypes of African elephants were country specific or even detected at only a single locality; and found that in a majority of cases where nuclear microsatellite alleles would mis-assign the origins of an elephant or its ivory, mtDNA could have contributed to a correct assignment. Thus, mtDNA appeared to be highly informative for establishing the provenance of elephants or their ivory. We considered whether a combination of mtDNA and nuclear markers would enhance the effectiveness of genetic assignment methods if applied to other species, in which low female and high male dispersal has led to distinct evolutionary trajectories for mitochondrial and nuclear markers.

## Materials and methods

### Samples

**Figure 1:**
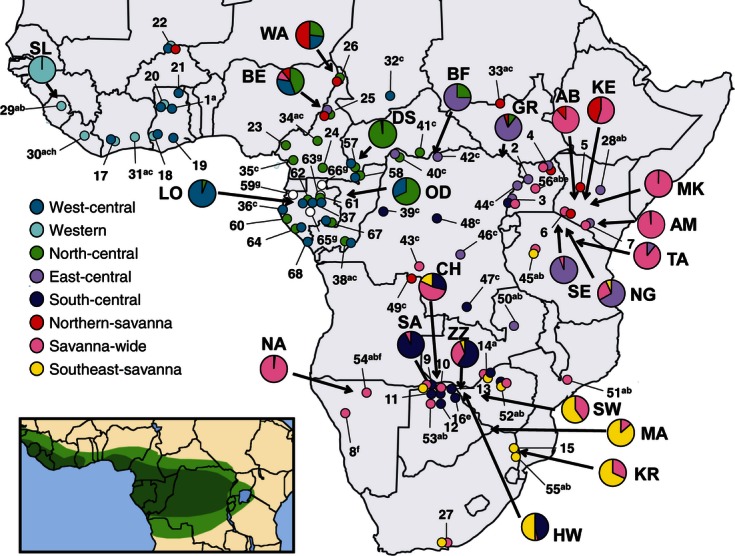
Map showing the geographic distribution of elephant mtDNA subclades across Africa. African elephant samples were grouped geographically into the localities represented by the large pie charts, which show the frequency at each of the localities of the eight major mtDNA subclades (Fig. 2) identified by this study. The small circles represent subclades present at locations sampled by previous mtDNA studies (Barriel et al. 1999; Eggert et al. 2002; Nyakaana et al. 2002; Debruyne et al. 2003; Debruyne 2005; Johnson et al. 2007), which were assigned using diagnostic sites for subclades (Fig. S1, Table S2). The inset map shows tropical forest (dark green) and mixed (light green) habitat zones (White 1983), which correspond approximately to the range of the forest elephant. Locations sampled by previous studies are numbered as in Ishida et al. (2011b) and are also listed in Table S7. White circles indicate that published information was insufficient regarding which mtDNA haplotype data corresponded to specific sampling locations (Johnson et al. 2007). Our sampling locations in tropical forest habitats were as follows: DS, Dzanga Sangha, Central African Republic; OD, Odzala, Republic of Congo; BF, Bili Forest, Democratic Republic of Congo; LO, Lope, Gabon; and SL, Sierra Leone (one zoo individual). Savanna locations: CH, Chobe, MA, Mashatu; SA-Savuti in Botswana; BE, Benoue; WA, Waza in Cameroon; AB, Aberdares; AM, Amboseli; KE, Central Kenya/Laikipia; MK, Mount Kenya in Kenya; NA, Northern Namibia/Etosha; KR, Kruger in South Africa; NG, Ngorongoro; SE, Serengeti; TA, Tarangire in Tanzania; and HW, Hwange; SW, Sengwa, ZZ, Zambezi in Zimbabwe. GR, Garamba is located in the Guinea-Congolian/Sudanian transition zone of vegetation in D.R. Congo that historically included a mixture of forest and secondary grasslands (White 1983) suitable for both African elephant groups (Groves and Grubb 2000). Subclade frequencies for BF were derived from control region sequences published by Ishida et al. (2011b) and were not included in some analyses.

The study was conducted under the University of Illinois Institutional Animal Care and Use Committee (IACUC)-approved protocol number 09036. Samples were collected in full compliance with required Convention on International Trade in Endangered Species of Wild Fauna and Flora and other institutional permits. About 700 wild African elephants (*Loxodonta*) were sampled, primarily by biopsy darting as previously described (Karesh et al. 1989; Georgiadis et al. 1994; Roca et al. 2002) (Fig. 1, Table S1). Asian elephant (*Elephas maximus*) DNA was extracted from blood samples generously provided by the Rosamond Gifford Zoo, Syracuse, NY, USA (Asian Elephant North American Regional Studbook No. 27, our designation ‘EMA 6’, a wild-caught male of unspecified origin), and the Oregon Zoo, Portland, OR, USA (Studbook No. 519, our designation ‘EMA 25’, a female from Borneo). DNA was purified primarily using DNA extraction kits from Qiagen (Valencia, CA); phenol–chloroform extraction was performed for some samples (Sambrook et al. 1989).

### PCR and sequencing

PCR was performed for two overlapping regions of mtDNA that together included a contiguous 4256–4258 bp of mtDNA sequence, which extended from part of *MT-ND5* to part of the control region. The first PCR used primers ND56-F1d and ND56-R1A (Brandt et al. 2012) and amplified 2568–2570 bp covering part or all of *MT-ND5*, *MT-ND6*, *MT-TE*, and *MT-CYTB*. The second PCR used primers CBCR-F1d and CBCR-R1d (Brandt et al. 2012) and amplified 1893–1895 bp covering all or part of *MT-TE*, *MT-CYTB*, *MT-TT*, *MT-TP*, and control region. The mitochondrial gene abbreviations used here follow recommendations of the HUGO Gene Nomenclature Committee (http://www.genenames.org/). The primers used for PCR or sequencing have been published elsewhere (Brandt et al. 2012). PCR used 0.4 μm final concentration of each oligonucleotide primer in 1.5 mm MgCl_2_, 200 μm of each of the dNTPs (Applied Biosystems Inc. [ABI], Foster City, CA), and 1× PCR Buffer II (ABI) with 0.04 units/μL final concentration of AmpliTaq Gold DNA Polymerase (ABI). For DNA that was of poor quality, 0.8 μg/μL final concentration of BSA (New England BioLabs Inc., Ipswich, MA) was included. PCR consisted of an initial 95°C for 9:45 min; with cycles of 20 s denaturing at 94°C, followed by 30 s annealing at 60°C (three cycles); 58, 56, 54, or 52°C (five cycles each temperature); or 50°C (last 22 cycles), followed by 3 min extension at 72°C; with a final extension of 7 min at 72°C. For samples of poor DNA quality, other primer combinations (Brandt et al. 2012) were used to generate shorter amplicons, with extension times changed to at least 1 min/1000 bp. We removed primers and unincorporated dNTPs from the PCR products with exonuclease I (USB Corporation, Santa Clara, CA) and shrimp alkaline phosphatase (USB Corporation) prior to sequencing (Hanke and Wink 1994). Sequences in both directions were generated using the BigDye Terminator v3.1 Cycle Sequencing Kit (ABI) with 2.5 μL of purified PCR products and 0.12 μm primer, as previously described (Ishida et al. 2011a), and purified and resolved on an ABI 3730XL capillary sequencer at the Core DNA Sequencing Facility of the University of Illinois at Urbana-Champaign. The software Sequencher 4.5 (Gene Codes Corp., Ann Arbor, MI) was used to edit chromatograms, assemble contigs for each amplicon, and concatenate the two sequenced regions of mtDNA; gene identity was established by homology to the reference savanna elephant genome sequence (GenBank No: NC_000934) (Hauf et al. 2000). The possibility of amplifying numts was minimized as previously described (Roca et al. 2007; Brandt et al. 2012).

### Phylogenetic analyses

Sequences were aligned with Clustal X version 2.0 (http://www.clustal.org/) (Larkin et al. 2007), with alignments visually inspected. Phylogenetic relationships among unique mtDNA haplotypes were inferred using four approaches implemented in PAUP* 4.0b10 (Altivec) (Swofford 2002). Maximum parsimony (MP) analysis was performed using a heuristic search, with nearest neighbor interchange (NNI) branch swapping. Model selection was conducted using the Akaike information criterion (AIC) implemented in Modeltest Version 3.06 (Posada and Crandall 1998); the HKY+I+G model was estimated to be the model that best fit our dataset, with base frequencies of A = 0.3345, C = 0.2772, G = 0.1110, and T = 0.2772, Nst (number of substitution types listed in a rate matrix) = 2, Ti/Tv ratio = 32.1897, rates (distribution of rates at variable sites) = gamma, shape (gamma distribution shape parameter) = 1.0311, and pinvar (proportion of invariant sites) = 0.6779. Neighbor Joining (NJ), minimum evolution (ME), and maximum likelihood (ML) analyses were implemented using this model. ME and ML employed heuristic searches with NNI branch swapping. Support for the nodes in each analysis was assessed by 100 bootstrap iterations.

### Assignment of previously published sequences to subclades

We examined polymorphisms across our sequences within 316 bp of the control region (Table S2) that were homologous to sequences previously generated by studies that examined trans-national mtDNA patterns in African elephants (Eggert et al. 2002; Nyakaana et al. 2002; Debruyne et al. 2003; Debruyne 2005; Johnson et al. 2007). A flow chart was made based on these polymorphic sites to assign the 316 bp of control region sequences to subclades (Fig. S1). Three Bili Forest (BF) elephants (GenBank: JF827273) had sequences missing 66 bp of the 3′-end of the 316 bp control region sequence, but they were assignable to a subclade using the available nucleotide sites. A distinct RGB color code was used to represent each mtDNA subclade, as follows: western (R: 161, G: 255, B: 211), west-central (R: 49, G: 136, B: 153), north-central (R: 102, G: 255, B: 0), east-central (R: 163, G: 128, B: 229), south-central (R: 0, G: 0, B: 255), northern savanna (R: 254, G: 0, B: 0), savanna-wide (R: 255, G: 111, B: 207), and southeast savanna (R: 255, G: 255, B: 0). RGB color codes were also used for nuclear partitions between forest elephants (R: 7, G: 80, B: 8) and savanna elephants (R: 248, G: 143, B: 0).

### Median-joining network and nuclear partitioning

Median-joining (MJ) networks were constructed using the software NETWORK 4.5.1.6 (http://www.fluxus-technology.com/sharenet.htm) (Bandelt et al. 1999). For our mtDNA sequences (4258 bp), haplotype frequencies were shown on the network. A separate MJ network was constructed combining our control region sequences with control region sequences (316 bp) generated by previous trans-national studies of African elephant mtDNA (Eggert et al. 2002; Nyakaana et al. 2002; Debruyne et al. 2003; Debruyne 2005; Johnson et al. 2007), including mtDNA sequences from elephants in the BF (Ishida et al. 2011b). It should be noted that as the fast-evolving control region is known to be unreliable as a molecular clock (Ingman et al. 2000), the deeper relationships across haplogroups, reliably established using the full alignment, would not necessarily be recapitulated using only the control region. Haplotype frequencies were not shown for the control region network that combined previous results because for some studies (Eggert et al. 2002) haplotype frequencies had not been reported. The mtDNA *F*_ST_ was calculated for each pair of localities using Arlequin ver. 3.11 (Excoffier et al. 2005), with mtDNA *F*_ST_ *P* values calculated using 10 000 permutations. To examine nuclear genetic partitions, the program *Structure* 2.3.1 (Pritchard et al. 2000) was run for 1 million Markov chain Monte Carlo generations following a burn-in of at least 100 000 steps, using a previously published microsatellite dataset (Ishida et al. 2011b), with the data re-organized by mtDNA subclade for the current analyses.

## Results

### Phylogeny of African elephant mtDNA

Samples were grouped into four tropical forest localities known to harbor *Loxodonta cyclotis* (forest elephants, *n* = 75) and 17 localities outside the tropical forest known to harbor *L. africana* (savanna elephants, *n* = 558) (Fig. 1). We also sequenced 20 elephants from Garamba, a locality that historically included a mix of both forest and savanna habitats (White 1983) and is home to both species of African elephant (Groves and Grubb 2000) including hybrids (Roca et al. 2001, 2005; Comstock et al. 2002). A total of 653 African elephant individuals were successfully sequenced for 4256 to 4258 bp of contiguous mtDNA sequence, at positions 11 750–16 006 in the African elephant reference mtDNA genome (Hauf et al. 2000), and all sequences were deposited in GenBank (accession numbers: JQ438119–JQ438771). We identified 424 variable sites and 108 haplotypes in African elephants. Table S1 lists the number of samples sequenced and haplotypes present for each locality.

**Figure 2:**
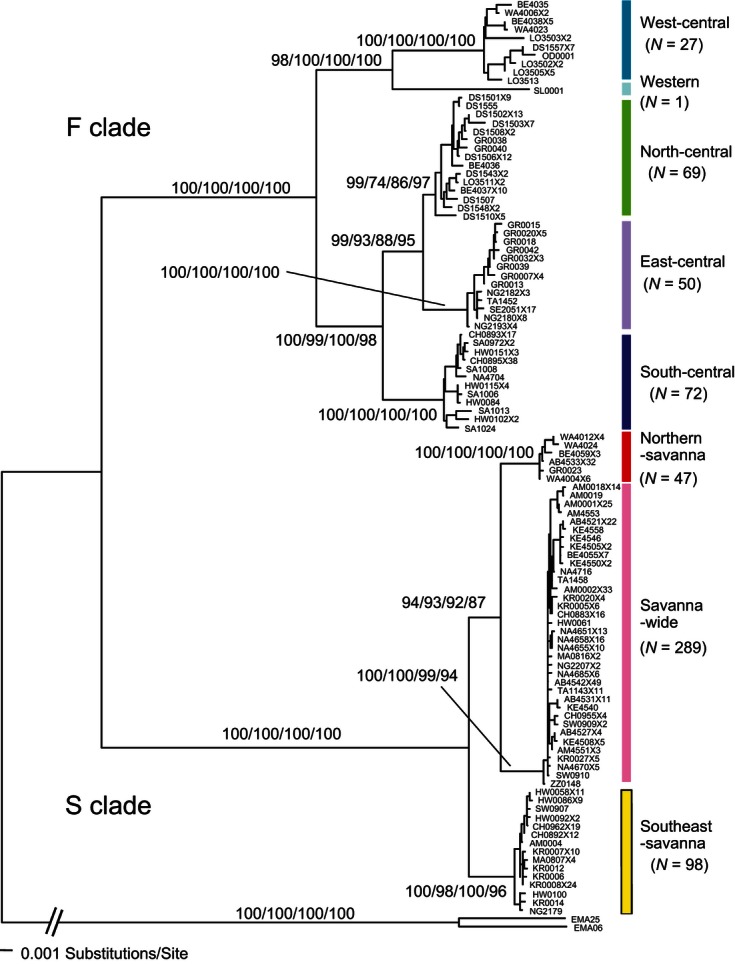
Phylogeny of a 4258-bp alignment supports the subdivision of mtDNA into eight major subclades. The alignment consisted of unique haplotypes found among 653 African elephants, comprising a continuous mtDNA sequence from part of *MT-ND5* to part of the control region. The phylogeny confirmed a deep split between S clade mtDNA (with three subclades), which is derived from and carried only by savanna elephants, and F clade mtDNA (with five subclades), which originates in forest elephants but is also carried by many savanna elephants (Roca and O'Brien 2005; Ishida et al. 2011b). The eight highly supported subclades were named for their regional distributions, as determined by this analysis and by assignment of sequences from previous studies (Fig. 1). The neighbor joining (NJ) tree is shown; bootstrap support for the major subclades is shown for (left to right) maximum parsimony, NJ, minimum evolution, and maximum likelihood. The first two letters in the designation of each elephant represents the location as abbreviated in Fig. 1 and Table S1. Only one individual was listed when more than one elephant shared identical haplotypes, although the number of individuals sharing the same haplotype is indicated (e.g., ‘X2’ indicates that the haplotype was present in the individual listed and in one other elephant). The tree was rooted using two Asian elephants (code: EMA).

Phylogenetic analyses of the 4258-bp mtDNA alignment using MP, neighbor joining (NJ), ME, and ML approaches produced trees with similar topologies (Fig. 2). As expected, the deepest subdivision detected in our phylogenies corresponded to the basal ‘F’ and ‘S’ mtDNA clades (Debruyne 2005), and the mtDNA pattern was nonmonophyletic for forest and savanna elephant species (Debruyne 2005; Roca et al. 2005; Lei et al. 2009). We detected S clade mtDNA only in savanna elephants (*n* = 433) and in one hybrid elephant from Garamba (GR0023), while F clade mtDNA was carried by all forest elephants (*n* = 75), the rest of the Garamba individuals (*n* = 19), and also by many savanna elephants (*n* = 125), in accordance with established patterns (Debruyne 2005; Roca et al. 2005; Lei et al. 2008; Ishida et al. 2011b). High bootstrap support was found for the further subdivision of F clade into five subclades and of S clade into three subclades (Fig. 2). The eight subclades detected in our dataset were greater in number than the 2–5 subdivisions previously reported for African elephant mtDNA (Eggert et al. 2002; Debruyne 2005; Roca et al. 2005; Johnson et al. 2007; Lei et al. 2008), likely due to the lengthy sequences, which provided a large number of informative sites and high resolution. Subclade-specific diagnostic sites are listed in Table S3.

### Phylogeographic distribution of mtDNA subclades

We assigned mtDNA sequences previously generated by trans-national studies of African elephants (Eggert et al. 2002; Nyakaana et al. 2002; Debruyne et al. 2003; Debruyne 2005; Johnson et al. 2007) to the 8 subclades using 316 bp of control region that overlapped across these studies and with our novel sequences. Among our sequences, the control region included 42 polymorphisms, of which two were S or F clade-specific diagnostic sites and five were subclade-specific diagnostic sites (Table S2). Considering only haplotypes within the F clade, an additional 19 subclade-specific polymorphisms were present, of which five were fixed by subclade (Table S2). For haplotypes within the S clade, an additional 11 subclade-specific polymorphisms were found, and four were fixed by subclade (Table S2). Based on these polymorphic sites, previously generated sequences could be assigned to the subclades, following the flowchart shown in Fig. S1. The accuracy of assignment of control region sequences to the eight subclades was further supported by the grouping of control region haplotypes within haplogroups on a network and by the continuous geographic distribution of the subclades. Using our own and previously generated sequences (Eggert et al. 2002; Nyakaana et al. 2002; Debruyne et al. 2003; Debruyne 2005; Johnson et al. 2007), the geographic distributions of the eight subclades across Africa were established (Fig. 1). We named subclades by their regional distributions within the African continent, with ‘savanna’ added to designations of the three subclades of the S clade, because S clade haplotypes were never carried by forest elephants.

The phylogenetic relationships among subclades were inferred using our sequences (Fig. 2). The most basal lineage within S clade, the ‘southeast savanna’ subclade, included haplotypes carried by most elephants in Mashatu, Sengwa, and Kruger (see Fig. 1 and Table S1 for the location abbreviations) and was distributed across much of southeast Africa at least as far north as Tanzania (Figs 1 and 2). The remaining S clade elephants included the ‘northern savanna’ subclade, which was present from Kenya north to the Sahelian/Sudanian savanna belt as far west as Mali (with one site in northern Angola, which may be a remnant of the glacial retreat of tropical forests in Central Africa) (Figs 1 and 2). Finally, the ‘savanna-wide’ subclade was the only subclade demonstrating a distribution across Africa rather than a regional distribution, although it was not present in tropical forest locations (Figs 1 and 2). The savanna-wide subclade was found in the savanna belts immediately south of the Sahara, at least as far west as Cameroon, as well as across eastern and southern Africa. ‘Savanna-wide’ subclade mtDNA haplotypes were carried by a majority of elephants in Kenya and Namibia.

Five subclades were highly supported within the F clade. The deepest divergence split the F clade into a group consisting of the western and west-central subclades, and a group consisting of south-central, east-central, and north-central subclades (Fig. 2). The west-central subclade haplotypes were carried by elephants in West Africa, in the Guinean forest block at least as far west as Cote d'Ivoire, as well as in Central Africa, in the Congolian forest block, primarily west of the Congo River (Fig. 1). The west-central subclade predominated among elephants in Lope. West-central subclade haplotypes were also present in savanna populations in Mali, Cameroon, and Chad. The sister subclade to the west-central subclade was designated the western subclade, as it was limited to populations west of the Dahomey/Benin gap (Fig. 1). Our own dataset included only a single individual sampled from this subclade, a zoo sample from an elephant that had originated in Sierra Leone (SL0001 in Fig. 2). We could be confident that this was a highly supported subclade, not just based on the deep separation of SL0001 mtDNA from that of individuals in the west-central subclade, but from previous reports that had compared this elephant's mtDNA sequence (Barriel et al. 1999) to other elephants regionally and convincingly reported high support for including SL0001 in a distinctive western mtDNA clade (Eggert et al. 2002; Debruyne et al. 2003; Debruyne 2005). In West Africa, forest elephants (identified as *L. cyclotis* by nuclear genotyping) have been found in savanna habitats in northern Ghana (Wasser et al. 2004), and these carried mtDNA of the western subclade (Fig. 1). Additionally, savanna elephants (*L. africana*) in West Africa also carried F clade haplotypes of the western subclade, as far north as the elephants of Mali.

Three other, related, subclades were present within the F clade (Fig. 2). The most basal of these three was the south-central subclade, which among our samples was carried by elephants in savanna populations and predominated in some populations as far south as Botswana and Zimbabwe (Fig. 1). Assignment of previously sequenced haplotypes found this subclade to be present in the Democratic Republic of Congo south of the Congo River, among elephants in both savanna and forest habitats, and east as far as the border regions of Uganda. The east-central subclade was found primarily east of the Congo River and was the predominant subclade among forest elephants of the BF and in the mixed population of Garamba (Fig. 1). It was also found in savanna populations from Cameroon east to Uganda, Kenya, and Tanzania, and as far south as Northern Zambia. It was the most common mtDNA subclade among savanna elephants in Serengeti and Ngorongoro. The north-central subclade was found primarily in the western part of the Congolian forest block and was the predominant subclade in Dzanga Sangha; though, it appeared to be absent from West Africa (Fig. 1). It was found at least as far to the east as Garamba and was carried by savanna elephants in Cameroon.

### Network analyses and mito-nuclear comparisons

**Figure 3:**
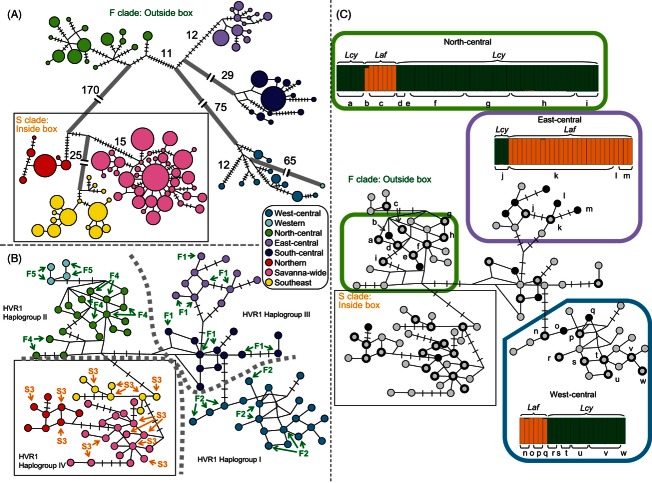
Mitochondrial and mito-nuclear patterns among African elephants. (A) Median-joining (MJ) network (Bandelt et al. 1999) generated using 4258-bp mtDNA sequences from our samples. Circle sizes are proportionate to the haplotype frequency. Each subclade is colored as in Fig. 1, with S clade haplotypes within the boxed region and F clade haplotypes outside. The number of nucleotide differences is indicated (if > 1) by hatch marks or by a number. (B) MJ network of 316-bp mtDNA, combining our control region sequences with those of previous trans-national elephant surveys (Barriel et al. 1999; Eggert et al. 2002; Nyakaana et al. 2002; Debruyne et al. 2003; Debruyne 2005; Johnson et al. 2007; Ishida et al. 2011b). Arrows indicate five haplotype subclades identified by Debruyne, while dotted lines indicate the four groups identified by Johnson et al. (Barriel et al. 1999; Eggert et al. 2002; Debruyne et al. 2003; Debruyne 2005; Johnson et al. 2007). (C) The overlap between our and previously identified haplotypes based on the 316-bp control region is shown. Novel haplotypes found by this study are filled black circles. Haplotypes detected by both the current and previous studies are thick outlined circles. Haplotypes reported only by previous studies are light gray circles. For three mtDNA subclades that included both forest and savanna elephants in our dataset, nuclear DNA partitions (*Structure K* = 2, orange and dark green) followed savanna (*Laf*) or forest (*Lcy*) species boundaries and did not partition by mtDNA subclade.

We generated a MJ network of elephant mtDNA using the 4258-bp alignment, finding that the haplotypes corresponding to each of the subclades in the phylogeny (Fig. 2) also grouped together in the network (Fig. 3A). We also generated a second network based only on 316 bp of control region, to display the degree of overlap between our mtDNA haplotypes and those generated by previous trans-national surveys of African elephant mtDNA (Eggert et al. 2002; Nyakaana et al. 2002; Debruyne et al. 2003; Debruyne 2005; Johnson et al. 2007). Haplotypes in the network corresponding to each of the subclades were color coded in Fig. 3. The fast-evolving control region is known to be unreliable as a molecular clock (Ingman et al. 2000); thus, the deeper relationships across haplogroups established using 4258 bp of mtDNA (Fig. 3A) were not always recapitulated using only the control region (Fig. 3B). Nonetheless, the 316-bp control region contained subclade diagnostic sites sufficient for each haplotype on the network to be assigned to a subclade, and the haplotypes assigned to each subclade grouped together on the network (Fig. 3B).

Figure 3B indicates how the eight African elephant subclades identified by our longer sequencing of mtDNA relate to five subclades previously identified by Debruyne (arrows labeled F1, F2, S3, F4, F5) (Debruyne 2005) and to four groups identified by Johnson et al. (2007) labeled HVR1 I–IV), 2007which combined data from various other studies (Eggert et al. 2002; Nyakaana et al. 2002; Debruyne et al. 2003; Debruyne 2005). Haplotype frequencies have not always been reported by previous studies (Eggert et al. 2002) and thus were not shown on the control region network. The distribution of our mtDNA haplotypes on the MJ network largely overlaps the distribution of the previously identified haplotypes (Fig. 3C). Thus, neither geographic sampling bias nor limited sample size prevented the samples of elephants in our study from encompassing much of the mtDNA diversity present across African elephants (Fig. 3C).

All forest and many savanna elephant individuals carried mtDNA that fell within the F clade. As each of the five subclades of the mtDNA F clade included both forest and savanna elephants, we sought to examine whether nuclear DNA partitions would occur by mtDNA subclade, or alternatively whether the elephants would show partitioning between forest and savanna elephants regardless of mtDNA subclade (Roca et al. 2005; Lei et al. 2008, 2009; Ishida et al. 2011b). We examined nuclear partitions among elephants within the mtDNA subclades for which we had both forest and savanna elephant samples: the north-central, east-central, and west-central subclades. Although western and south-central subclade haplotypes are also carried by both forest and savanna elephant populations (Fig. 1), our own geographic sampling included only one of the species carrying these haplotypes. As S clade is not carried by forest elephants (Ishida et al. 2011b), the three subclades within S clade were not considered (Figs 1 and 2). For the three mtDNA subclades for which our dataset included both forest and savanna elephants, nuclear DNA partitions were determined, using *Structure* to analyze previously generated microsatellite genotypes (Pritchard et al. 2000; Ishida et al. 2011b). We found that nuclear genotypes did not partition by mtDNA subclade. Instead, within each subclade, partitioning occurred between forest and savanna elephants (Fig. 3C). For the three mtDNA haplogroups identified by Johnson et al. (2007) as including both forest and savanna elephants (Fig. 3B), our results (Fig. 3C) affirm and extend to the subclade-level previous reports that the phylogeography of mtDNA and nuclear genotypes are incongruent among African elephants (Roca et al. 2005; Lei et al. 2008, 2009; Ishida et al. 2011b).

### Potential of African elephant mtDNA for identifying the provenance of ivory

**Figure 4:**
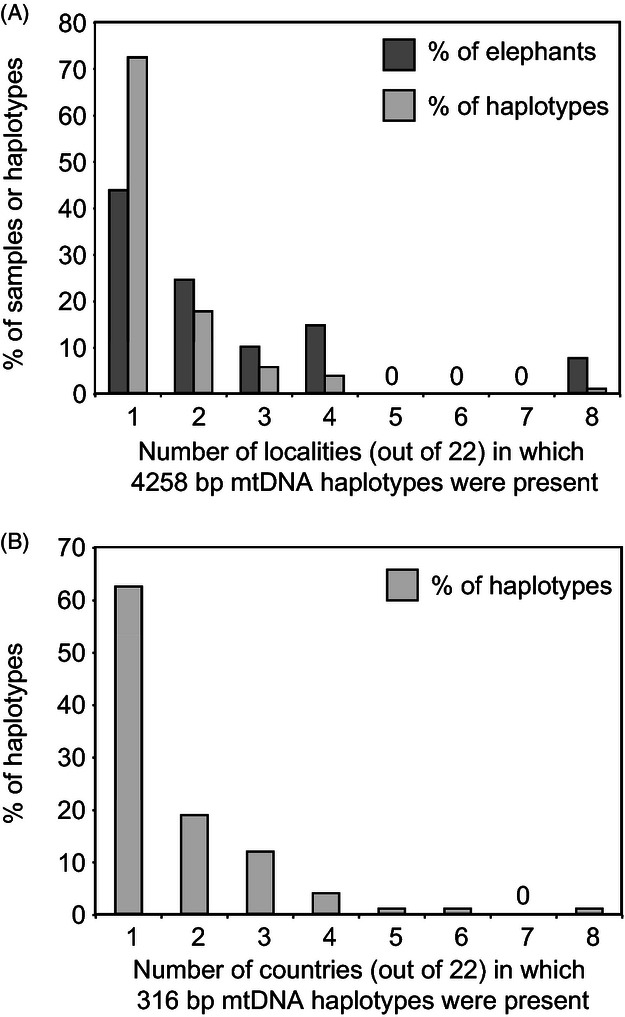
Geographic distribution of African elephant haplotypes (A) Mitochondrial DNA sequences (4258 bp, from *MT-ND5* to control region) were generated from 653 African elephants from 22 localities, with 108 unique haplotypes identified. The *x*-axis indicates whether haplotypes were detected in one or in multiple geographic localities. The *y*-axis indicates (in light shading) the percentage of unique haplotypes that were found to be carried by elephants across a given number of localities (from 1 to 8). The darker shading indicates the proportion of elephant individuals carrying haplotypes present in the number of localities indicated. It should be noted that 72% of the haplotypes were found at only a single locality, with 44% of elephant individuals carrying locality-specific haplotypes. (The number of haplotypes found across 5, 6 or 7 localities was zero.) (B) A 316-bp sequence of African elephant mtDNA control region from our dataset was combined with the sequencing results of previous trans-national datasets (Eggert et al. 2002; Nyakaana et al. 2002; Debruyne et al. 2003; Debruyne 2005; Johnson et al. 2007). The distribution of 101 unique haplotypes from 22 countries is shown; in this case, the *x*-axis indicates number of countries rather than localities. The *y*-axis indicates the number of unique haplotypes found to be geographically distributed across one or more countries. It should be noted that 62% of haplotypes were detected in a single country. (The number of haplotypes found across seven countries was zero.)

We examined whether the distinctive phylogeographic signals provided by mtDNA in elephants could be used to infer the geographic origins of confiscated ivory. We defined a unique haplotype as referring to a distinct mtDNA sequence, which may be carried by one or more elephants. We determined whether each unique mtDNA haplotype was present among elephants at a single locality, at more than one locality within a single country, or across elephants from more than one country. Among the 4258-bp mtDNA haplotypes, 72% of unique haplotypes proved to be locality specific, and 44% of individual elephants carried these locality-specific haplotypes (Table S4, Figs 4A and S2A). Among the 22 localities into which our samples were grouped, there were 20 localities in which some elephants carried locality-specific haplotypes not detected among elephants at any other localities (Table S4, Fig. S2A). Additionally, among our samples, 84% of unique haplotypes were country specific and 66% of the elephants carried country-specific haplotypes (Table S4).

We also examined the geographic distribution of the control region sequence common to our data and to previous trans-national studies (Eggert et al. 2002; Nyakaana et al. 2002; Debruyne et al. 2003; Debruyne 2005; Johnson et al. 2007). Being only 316 bp, we would expect that this short control region segment would be readily amplified and sequenced in DNA from ivory (Mailand and Wasser 2007). Across 22 countries (Fig. 1), 101 unique haplotypes were identified (Fig. 3). Among the 101 haplotypes, 63 (62%) were detected only in a single African country (Table S5, Figs 4B and S2B). Thus, a majority of mtDNA haplotypes showed very restricted geographic distributions within Africa. Among 22 countries surveyed, there were 17 countries in which some of the elephants carried country-specific haplotypes not detected in any other countries (Table S5, Fig. S2B).

**Figure 5:**
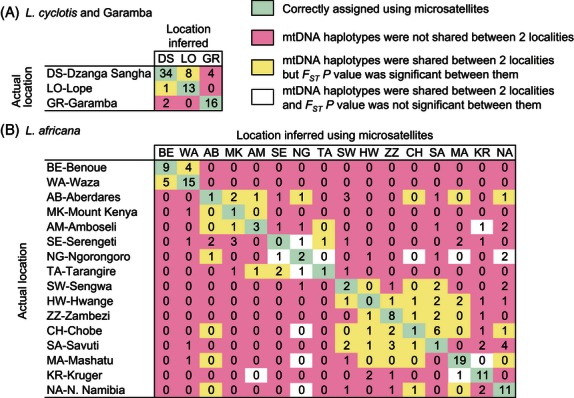
Methods to establish the provenance of ivory are enhanced by the addition of mtDNA data. The tables shown are based on the results of geographic assignments based on microsatellite genotypes conducted by Wasser et al. (2004) for localities and samples that overlap those of this study. The actual origin of the elephants is shown along with the provenance estimated using microsatellite markers for (panel A) forest elephant localities and (panel B) savanna elephant localities. Light green color indicates that 148 samples were correctly assigned by the microsatellite markers. For 55 of 122 samples that were mis-assigned by microsatellites, mtDNA haplotypes were not shared between the actual and estimated localities (rose shading), and mtDNA sequence data would have precluded the mis-assignment. For an additional 60 cases of mis-assignment (yellow shading), although mtDNA haplotypes were shared between the actual and inferred localities, a significant mtDNA *F*_ST_ value was calculated between the two localities, suggesting that frequencies of mtDNA haplotypes could be used to distinguish between them. Additionally, the presence of locality-specific mtDNA haplotypes (see Tables S4, S5, Figs 4 and S2) among batches of ivory would further preclude mis-assignments. Mis-assignments that were not further resolved with mtDNA haplotypes are unshaded.

Microsatellite DNA markers have been applied previously to identify the origin of smuggled ivory (Wasser et al. 2004, 2007, 2008), using as voucher specimens many of the same elephants sequenced for this study. Given that mtDNA provides a phylogeographic signal distinctive and sometimes orthogonal to the pattern of nuclear DNA (Fig. 3C) (Roca et al. 2005; Lei et al. 2008, 2009; Ishida et al. 2011b), we examined the degree to which mtDNA would enhance the accuracy of nuclear DNA assignments of the provenance of elephants (or their ivory). To do this, we turned to the results of a microsatellite assignment study published by Wasser et al. (2004), which overlapped with our own study in terms of samples used and localities examined. For the locations that were also part of our own study, they had reported that for 148 of 270 elephants, the geographic assignment of elephants using microsatellites matched the actual provenance of the elephants. They also reported 122 cases (15 of 78 for forest and 107 of 192 savanna elephants), in which the provenance was mis-assigned to an incorrect location (Wasser et al. 2004). We considered whether the use of mtDNA sequences from elephants at these locations would help to assign their provenance. We found that in 55 of the 122 cases of mis-assignment (six of 15 for forest and 49 of 107 for savanna elephants), there would have been no overlap in mtDNA haplotypes between the locality that was the actual source of the elephant sample and the locality to which it was wrongly assigned using microsatellites (Fig. 5). In these cases, sequencing of mtDNA would have precluded the elephants from being assigned to the wrong locality.

For many of the pairwise comparisons, the two localities had no haplotypes in common (Fig. 5). The lack of overlap in haplotypes between some localities was not attributable to inadequate sampling. Between the highly sampled southern African locations of Kruger (*n* = 50 elephants; nine unique haplotypes) and northern Namibia (*n* = 60; nine unique haplotypes), no haplotypes were shared between localities. While between Dzanga Sangha (*n* = 54; 11 unique haplotypes) and Lope (*n* = 17; six unique haplotypes), only one haplotype was shared between localities. Even across populations that shared mtDNA haplotypes, the high degree of geographic structuring among mtDNA haplotypes often led to significant mtDNA *F*_ST_ values between populations (Table S6). Although this is not reflective of nuclear population structure (Fig. 3C), it suggests that even for elephants from localities that shared some haplotypes, sets of elephants might be distinguished by differences in haplotype frequencies (Fig. 5, Table S6). Analyses of frequencies of haplotypes across individuals would be helpful for establishing the origins of ivory that was confiscated in batches, because assignment studies using nuclear markers have shown both that batches of ivory may derive from a single geographic source, and that batch analyses can improve the assignment of provenance (Wasser et al. 2007, 2008). In an additional 60 of the 122 cases of mis-assignment (nine for forest and 51 savanna elephants), a significant mtDNA *F*_ST_ difference was calculated between the actual locality and the locality to which an elephant was mis-assigned (Fig. 5). Overall, in 115 of 122 mis-assigned cases (15 of 15 for forest and 100 of 107 savanna elephants), mtDNA could have improved estimates of the provenance of elephants and their ivory.

## Discussion

Several aspects of mtDNA phylogeography contribute to its ability to establish the provenance of African elephants, whether by itself (Tables S4, S5, Figs 4 and S2) or in combination with nuclear markers (Fig. 5). First, the eight major mtDNA subclades are limited to various degrees in their geographic distributions (Fig. 1). S clade elephants would not come from the tropical forest and could be regionally assigned for two of three subclades (Fig. 1) (Ishida et al. 2011b), while an elephant carrying mtDNA from any of the subclades within the F clade would have originated within one of five geographically limited regions within Africa. African tropical forest habitats likely fragmented during glacial cycles into a set of discontinuous refugia (Mayr and O'Hara 1986; Maley 2001; deMenocal 2004). With the caveat that the locations and extent of Pleistocene refugia for African tropical forests remain controversial (Lowe et al. 2010), it is possible that each of the five subclades within the F clade may represent the allopatric isolation of ancestral forest elephants into a separate glacial forest refugium (Brandt et al. 2012).

Second, unique haplotypes were much more limited in geographic distribution than were the subclades to which they belong. Thus, for our 4258-bp dataset, and for the combined 316-bp control region dataset, 84% and 62% of haplotypes, respectively, were detected in only a single country (Tables S4, S5, Figs 4 and S2). Third, while increasing the sample size of the voucher collection might increase the number of haplotypes found to be shared between localities, the larger sampling would also tend to identify rare haplotypes. These rare haplotypes would tend to be restricted in geographic range and may be limited to a particular locality or country (Tables S4, S5, Figs 4 and S2) and would thus be especially informative for establishing the provenance of ivory. Finally, large differences in haplotype frequencies can persist between locations because mtDNA exhibits very low geographic dispersal. Most pairwise comparisons showed significant differences in haplotype distributions (mtDNA *F*_ST_) even across localities that shared one or more haplotypes (Fig. 5). In many cases, ivory is confiscated in batches (Wasser et al. 2007, 2008), and by testing multiple elephants, it would be possible to examine haplotype frequencies, as well as to identify haplotypes among the batches that are locality specific, to establish the origins of the ivory.

The groundbreaking and increasingly sophisticated analyses conducted by Wasser and colleagues have advanced efforts to identify the origins of smuggled ivory using nuclear DNA markers (Wasser et al. 2004, 2007, 2008). As mtDNA phylogeographic patterns are incongruent when compared to those of nuclear DNA markers (Fig. 3C) (Roca et al. 2005, 2007; Lei et al. 2008, 2009; Ishida et al. 2011b), microsatellite markers and mtDNA provide essentially independent information on the geographic origins of elephants. Thus, although mtDNA by itself is a powerful tool for establishing the provenance of elephants (Tables S4, S5, Figs 4 and S2), mtDNA combined with nuclear markers provides a means for genetically triangulating the geographic origin of confiscated ivory, increasing the accuracy and precision of geographic assignments (Fig. 5). The successful identification of the geographic sources of illegal ivory may in turn allow law enforcement and conservation efforts to focus on identified poaching hotspots.

Molecular genetic data are increasingly being used to assign wildlife or wildlife products to their species, subspecies, or population of origin (Baker 2008; Eaton et al. 2010; Luo et al. 2010). For example, Baker and colleagues used mitochondrial DNA to determine the species and population sources for whale products in Japan and South Korea (Baker et al. 2000), while genetic data have also been used to infer the provenance of wildlife products made from the teeth of wolves in Italy (Caniglia et al. 2010) and to identify the geographic origins of rescued chimpanzees in Cameroon (Ghobrial et al. 2010). For the current study, we determined that in elephants, mtDNA and nuclear analyses provided different phylogeographic information that could be combined to triangulate the origins of ivory. This results from the distinctive evolutionary trajectory followed by mtDNA (Roca 2008) because females do not migrate between herds, so that male elephants mediate nuclear gene flow between herds. Male-biased dispersal occurs in a majority of mammalian species (Lawson Handley and Perrin 2007). While more extreme in elephants than in many other mammals, nonetheless male-biased dispersal in other taxa would lead to greater substructure in mtDNA and could also result in incongruent mitochondrial and nuclear phylogeographic patterns (Petit and Excoffier 2009). Thus, combining mtDNA with nuclear markers may prove effective for triangulating the provenance of confiscated wildlife products from a range of endangered taxa (http://www.traffic.org).

## Data archiving statement

DNA sequences have been deposited in GenBank (accession numbers JQ438119–JQ438771).

## Supporting Information

**Figure S1.** Flowchart useful for assigning previously published 316 bp control region sequences to eight mtDNA subclades.

**Figure S2.** Geographic distributions of African elephant mtDNA haplotypes among countries or localities.

**Table S1.** Number of samples and number of unique haplotypes for each locality.

**Table S2.** Polymorphisms within 316 bp of the control region.

**Table S3.** Clade and subclade specific diagnostic sites (4258 bp).

**Table S4.** Geographic distribution of mtDNA haplotypes (4258 bp).

**Table S5.** Geographic distribution of control region haplotypes.

**Table S6.** Population pairwise *F*_ST_ and *F*_ST_ *P* values for mtDNA.

**Table S7.** List of sample locations (shown as numbers in Figure 1).
