# Supplementary material for: Triangulating the provenance of African elephants using mitochondrial DNA
Source: Evol Appl. 2012 Aug 1;6(2):253–65. doi: 10.1111/j.1752-4571.2012.00286.x (PMC3689351; doi:10.1111/j.1752-4571.2012.00286.x)
Supplement: Supplementary file 1 [file eva0006-0253-SD1.pdf]

Triangulating the provenance of African elephants using mitochondrial DNA

Yasuko Ishida*, Nicholas J. Georgiadis[†], Tomoko Hondo*[‡] and Alfred L. Roca*[§¶]

*Department of Animal Sciences, University of Illinois at Urbana-Champaign
Urbana, IL 61801 USA

[†]80 S. Lyter Ave., Port Townsend, WA 98368, USA

[‡]Current address: Japan Eco-Science (Nikkan Kagaku) Co. Ltd., Shiomigaoka-chou 11-1-2F,Chuuou-ku, Chiba 260-0034, Japan

[§]Institute for Genomic Biology, University of Illinois at Urbana-Champaign Urbana, IL 61801 USA

[¶]Corresponding author: Alfred L. Roca, Ph.D.
Email: roca@illinois.edu

Supporting information (SI)

SI References

Barriel V, Thuet E, Tassy P (1999) Molecular phylogeny of Elephantidae. Extreme divergence of the extant forest African elephant. *C R Acad Sci III* **322**, 447-454.

Debruyne R (2005) A case study of apparent conflict between molecular phylogenies: the interrelationships of African elephants. *Cladistics* **21**, 31-50.

Debruyne R, Van Holt A, Barriel V, Tassy P (2003) Status of the so-called African pygmy elephant (*Loxodonta pumilio* [NOACK 1906]): phylogeny of cytochrome b and mitochondrial control region sequences. *C R Biol* **326**, 687-697.

Eggert LS, Rasner CA, Woodruff DS (2002) The evolution and phylogeography of the African elephant inferred from mitochondrial DNA sequence and nuclear microsatellite markers. *Proc R Soc Lond B Biol Sci* **269**, 1993-2006.

Groves, Colin P., and Peter Grubb. 2000. Do *Loxodonta cyclotis* and *L. africana* interbreed? *Elephant* 2 (4):4-7.

Ishida Y, Oleksyk TK, Georgiadis NJ*, et al.* (2011) Reconciling apparent conflicts between mitochondrial and nuclear phylogenies in African elephants. *PLoS ONE* **6**, e20642.

Johnson MB, Clifford SL, Goossens B*, et al.* (2007) Complex phylogeographic history of central African forest elephants and its implications for taxonomy. *BMC Evol Biol* **7**, 244.

Nyakaana S, Arctander P, Siegismund HR (2002) Population structure of the African savannah elephant inferred from mitochondrial control region sequences and nuclear microsatellite loci. *Heredity* **89**, 90-98.

White F (1983) *The Vegetation of Africa* UNESCO, Paris.

Figure S1. Flowchart useful for assigning previously published 316 bp control region sequences to eight mtDNA subclades. The chart was made based on polymorphisms contained in the control region (Table S2) across 653 elephant sequences generated for the current study, which were divided into subclades based on 4258 bp mtDNA (Figure 2). Next to the arrows are character states useful for subclade assignment, with the positions of the nucleotides shown relative to the 4258 bp alignment generated for the current study (outside parentheses) and relative to the 316 bp sequence alignment of Johnson et al. (Johnson *et al.*, 2007) (within parentheses).

[Figure S1]

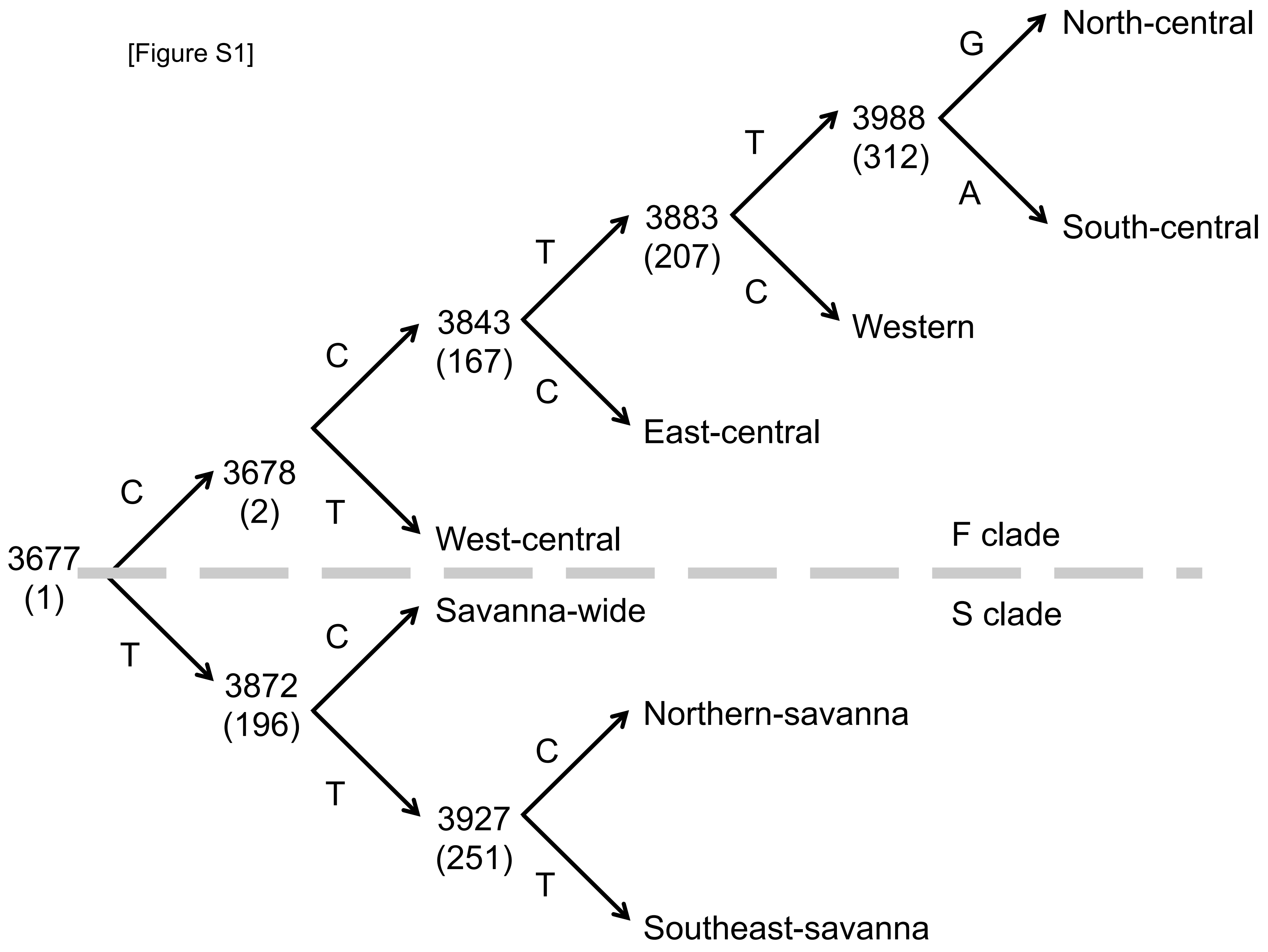

North-central
G
3988
(312)
T
A
South-central
3883
(207)
T
C
Western
3843
(167)
C
C
East-central
3678
(2)
C
T
West-central
F clade
3677
(1)
S clade
Savanna-wide
C
T
3872
(196)
Northern-savanna
C
T
3927
(251)
T
Southeast-savanna

Figure S2. Geographic distributions of African elephant mtDNA haplotypes among countries or localities. (A) For each locality, the number of elephant individuals sequenced for a 4258 bp segment of mtDNA is indicated. Shading indicates the number of individual elephants that carried haplotypes that were found only in a specific locality, or in larger numbers of localities. Among unique haplotypes present among elephants sequenced for the current study, 72% of the haplotypes proved to be locality-specific and 84% of haplotypes were country specific. Considering individual elephants, 44% of sampled elephants carried a locality-specific haplotype, and 66% a country-specific haplotype. The locality abbreviations are: DS-Dzanga Sangha, Central African Republic (CAR); OD-Odzala, Republic of the Congo (RC); BF-Bili Forest, Democratic Republic of the Congo (DRC); LO-Lope, Gabon; and SL-Sierra Leone (one zoo individual). Savanna locations: CH-Chobe, MA-Mashatu, SA-Savuti in Botswana; BE-Benoue, WA-Waza in Cameroon; AB-Aberdares, AM-Amboseli, KE-Central Kenya/Laikipia, MK-Mount Kenya in Kenya; NA-Northern Namibia/Etosha; KR-Kruger in South Africa; NG-Ngorongoro, SE-Serengeti, TA-Tarangire in Tanzania; HW-Hwange, SW-Sengwa, ZZ-Zambezi in Zimbabwe. GR-Garamba is located in the Guinea-Congolian/Sudanian transition zone of vegetation in D.R. Congo that historically included a mixture of forest and secondary grasslands (White 1983) suitable for both African elephant groups (Groves and Grubb 2000). (B) Combining 316 bp of African elephant mtDNA control region from the current study with those of previously published trans-national datasets (Eggert et al. 2002; Nyakaana et al. 2002; Debruyne et al. 2003; Debruyne 2005; Johnson et al. 2007), the distribution of unique haplotypes was examined. Among unique haplotypes, 62% were detected only in a single country. The frequencies of haplotypes by location had not

been reported by some studies, and thus could not be quantified for the control region analysis. Note that this accentuates haplotypes with broader distributions, e.g., haplotypes detected in just one country will be counted on only one bar of the chart, while the single haplotype found across 8 countries appears eight times.

[Figure S2]

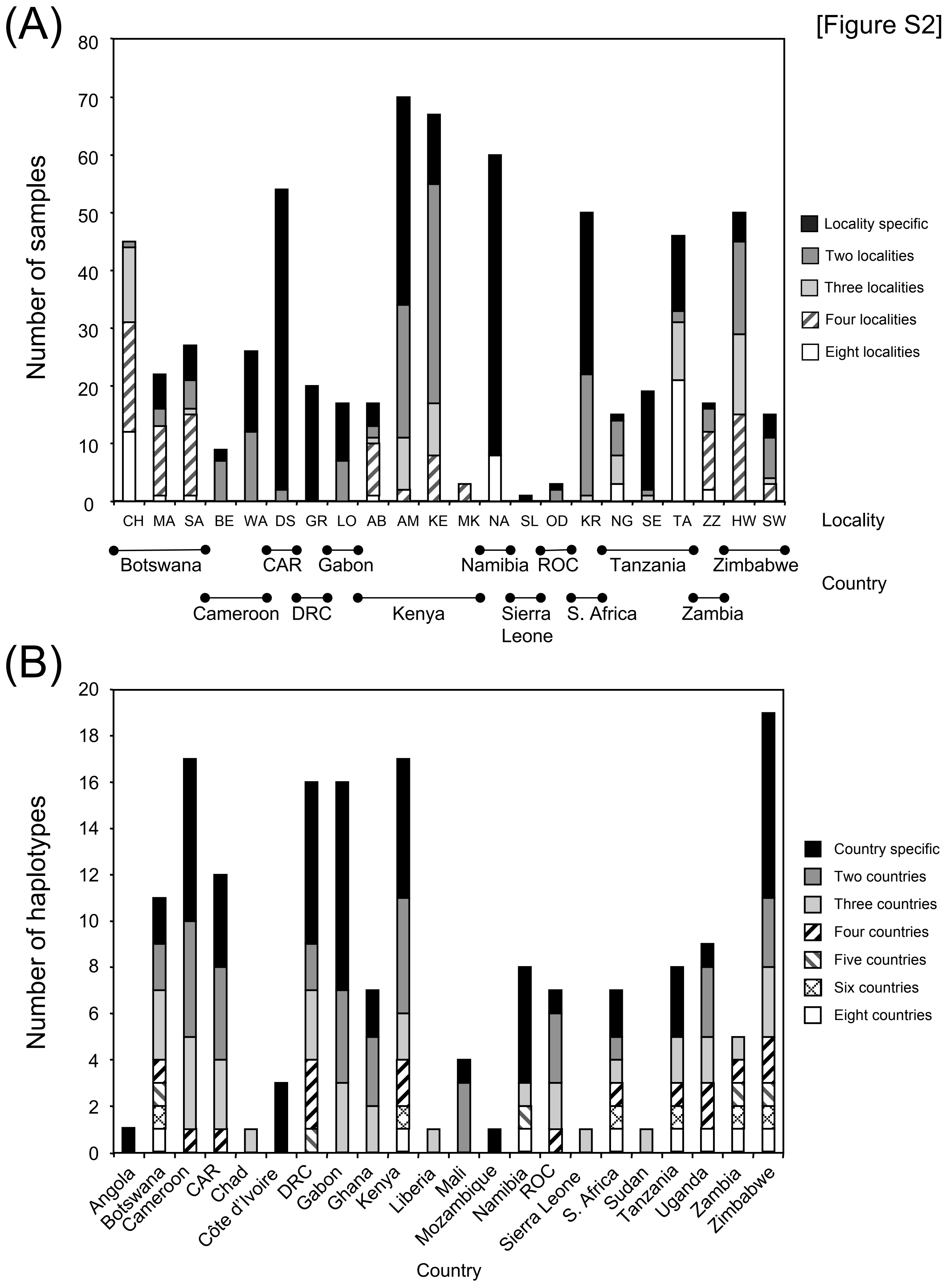

(A)
Number of samples
Locality specific
Two localities
Three localities
Four localities
Eight localities
CH MA SA BE WA DS GR LO AB AM KE MK NA SL OD KR NG SE TA ZZ HW SW
Locality
Botswana
CAR
Gabon
Namibia
ROC
Tanzania
Zimbabwe
Country
Cameroon
DRC
Kenya
Sierra Leone
S. Africa
Zambia
(B)
Number of haplotypes
Country specific
Two countries
Three countries
Four countries
Five countries
Six countries
Eight countries
Angola
Botswana
Cameroon
CAR
Chad
Côte d'Ivoire
DRC
Gabon
Ghana
Kenya
Liberia
Mali
Mozambique
Namibia
ROC
Sierra Leone
S. Africa
Sudan
Tanzania
Uganda
Zambia
Zimbabwe
Country

Table S1. Number of samples and number of unique haplotypes for each locality.

Species	*L. cyclotis*					*L. africana*																
Localities	DS	SL	LO	OD	GR	AB	AM	BE	CH	HW	KE	KR	MA	MK	NA	NG	SA	SE	SW	TA	WA	ZZ
Sample No.	54	1	17	3	20	17	70	9	45	50	67	50	22	3	60	15	27	19	15	46	26	17
Haplotype No.	11	1	6	3	12	5	9	7	8	14	13	9	5	1	9	6	11	3	7	8	8	6

Forest locations: DS-Dzanga Sangha, Central African Republic; OD-Odzala, Republic of the Congo; BF-Bili Forest (data from Ishida et al. (2011)), Democratic Republic of the Congo; LO-Lope, Gabon; and SL-Sierra Leone (one zoo individual). GR-Garamba in Democratic Republic of the Congo is in the Guinea-Congolian/Sudanian transition zone of vegetation that includes a mixture of forest and secondary grasslands (White, 1983) with both types of elephants. Savanna locations: CH-Chobe, MA-Mashatu, SA-Savuti in Botswana; BE-Benoue, WA-Waza in Cameroon; AB-Aberdares, AM-Amboseli, KE-Central Kenya/Laikipia, MK-Mount Kenya in Kenya; NA-Northern Namibia/Etosha; KR-Kruger in South Africa; NG-Ngorongoro, TA-Tarangire, SE-Serengeti in Tanzania; SW-Sengwa, HW-Hwange, ZZ-Zambezi in Zimbabwe.

Table S2. Polymorphisms within 316 bp of the control region.

Position* relative to:			F clade					S clade		
NC_000934	Current dataset (4258 bp)	Control region (316 bp)	North-central (69)	East-central (50)	South-central (72)	West-central (27)	Western (1)	Savanna-wide (289)	Northern-savanna (47)	Southeast-savanna (98)
15425 (del)	3676	1†	**A**	**A**	**A**	**A**	**A**	Δ	Δ	Δ
15425	3677	1	**C**	**C**	**C**	**C**	**C**	**T**	**T**	**T**
15426	3678	2	C	C	C	**T**	C	C	C	C
15429	3681	5	Y	C	C	C	C	C	C	C
15440	3692	16	**Y**	T	T	**Y**	**C**	T	T	T
15458	3710	34	**C**	T	**Y**	**Y**	**C**	T	T	T
15516	3768	92	Y	C	C	Y	T	Y	Y	T
15563	3815	139	T	Y	T	T	T	T	T	T
15573	3825	149	C	**Y**	**T**	**Y**	C	C	C	C
15574	3826	150	T	**Y**	**C**	**Y**	T	T	T	T
15580	3832	156	G	R	G	G	G	G	G	G
15591	3843	167	T	**C**	T	T	T	T	T	T
15594	3846	170	R	A	A	**C**	A	A	A	A
15601	3853	177	T	T	Y	T	T	T	T	T
15602	3854	178	T	T	T	Y	T	T	T	T
15606	3858	182	A	R	A	A	A	A	A	A
15608	3860	184	G	**A**	G	**A**	G	G	G	G
15610	3862	186	Y	C	C	C	C	Y	Y	C
15612	3864	188	T	Y	T	T	T	T	T	T
15616	3868	192	Y	C	C	Y	C	C	T	Y
15617	3869	193	T	T	T	T	T	T	Y	T
15618	3870	194	A	A	R	A	A	A	G	R
15619	3871	195	G	G	G	G	G	R	G	G
15620	3872	196	T	T	T	T	T	**C**	T	T
15621	3873	197	T	T	T	T	T	**Y**	**Y**	T
15631	3883	207	T	T	T	T	**C**	T	T	T
15632	3884	208	A	A	A	A	A	**G**	**G**	A
15635	3887	211	T	T	Y	C	T	C	C	T
15636	3888	212	A	A	**R**	**R**	A	A	A	A
15639	3891	215	T	T	T	T	T	**C**	**C**	T
15640	3892	216	A	A	A	A	A	R	A	A
15648	3900	224	C	C	C	Y	C	C	C	C
15675	3927	251	T	T	T	Y	T	T	C	T
15680	3932	256	A	A	A	A	A	R	A	G
15685	3937	261	A	A	A	A	A	A	R	A
15691	3943	267	C	C	C	C	**T**	C	C	C
15692	3944	268	R	R	A	A	G	R	A	A
15696	3948	272	T	T	T	T	T	T	C	C
15716	3968	292	**Y**	**Y**	**Y**	**Y**	**T**	C	C	C
15728	3980	304	C	C	**Y**	C	**T**	C	C	C
15735	3987	311	C	C	Y	C	C	T	T	T
15736	3988	312	G	**A**	**A**	**A**	G	G	G	G
15737	3989	313	R	G	G	G	G	G	G	G

*Three positions are indicated for nucleotides; the first position is relative to the *Loxodonta africana* complete mitochondrial genome (GenBank No: NC_000934); the second position is relative to our 4258 bp mtDNA alignment. The third position is based on 316 bp of control region from Johnson et al. (2007). †The position 0 refers to an indel present (or absent, if delta) in the nucleotide that is immediately 5' of position 1 as designated by Johnson et al. (2007). Within the region shown, only the first two nucleotide positions (in boldface and unshaded) were fixed between F clade and S clade haplotype sequences. Clade specific nucleotide polymorphisms that were found in more than one subclade are in black bold letters and lightly shaded. Subclade specific fixed nucleotide site character states within the F clade, or within the S clade, are in boldface white letters and darkly shaded. Subclade specific polymorphisms within the F or within the S clade are shown in bold white letters that have intermediate shading. Numbers in parentheses indicate our sample sizes for elephants for each subclade.

Table S3. Clade and subclade specific diagnostic sites (4258 bp).

Position* relative to:		F clade					S clade		
NC_000934	Current dataset (4258 bp)	North-central (69)	East-central (50)	South-central (72)	West-central (27)	Western (1)	Savanna-wide (289)	Northern-savanna (47)	Southeast-savanna (98)
11758	9	C	C	C	C	C	C	C	**T**
11777	28	T	T	T	T	T	T	**C**	T
11794	45	C	C	C	C	C	T	T	T
11796	47	A	A	A	A	**G**	A	A	A
11802	53	**C**	T	T	T	T	T	T	T
11832	83	T	T	T	T	T	C	C	C
11839	90	C	C	C	C	C	T	T	T
11876	127	C	C	C	C	C	T	T	T
11891	142	A	A	A	A	A	G	G	G
11949	200	T	T	T	T	T	C	C	C
11967	218	C	C	C	C	C	A	A	A
11975	226	T	T	T	**C**	T	T	T	T
11994	245	T	T	T	T	T	C	C	C
12083	334	C	C	C	C	C	T	T	T
12095	346	T	T	T	T	T	C	C	C
12099	350	C	C	C	**T**	C	C	C	C
12108	359	C	C	C	C	**T**	C	C	C
12125	376	C	C	C	C	C	T	T	T
12194	445	G	G	G	**A**	G	G	G	G
12224	475	C	C	**T**	C	C	C	C	C
12254	505	T	T	T	T	T	C	C	C
12263	514	T	T	T	T	T	C	C	C
12272	523	**C**	T	T	T	T	T	T	T
12284	535	T	T	T	T	T	**C**	T	T
12304	555	T	T	T	T	T	C	C	C
12320	571	C	C	C	C	C	T	T	T
12342	593	T	T	T	**C**	T	T	T	T
12347	598	T	T	T	T	T	**C**	T	T
12350	601	C	C	C	C	**T**	C	C	C
12356	607	G	G	G	G	**A**	G	G	G
12389	640	A	A	A	A	A	G	G	G
12410	661	C	C	C	**T**	C	C	C	C
12416	667	T	T	T	T	T	C	C	C
12443	694	T	T	T	T	T	C	C	C
12455	706	C	C	C	C	C	T	T	T
12458	709	C	C	C	C	C	T	T	T
12497	748	A	A	A	**G**	A	A	A	A
12500	751	C	C	C	C	C	T	T	T
12524	775	T	T	T	T	T	T	**C**	T
12527	778	T	T	T	T	T	C	C	C
12596	847	C	C	C	C	C	T	T	T
12623	874	C	C	C	C	C	T	T	T
12650	901	C	C	**G**	C	C	C	C	C
12662	913	A	A	A	A	**G**	A	A	A
12669	920	C	C	C	C	C	T	T	T
12683	934	C	C	C	**T**	C	C	C	C
12689	940	C	C	C	C	**T**	C	C	C
12698	949	A	A	A	A	A	G	G	G
12719	970	T	T	T	T	T	C	C	C
12725	976	T	T	T	**C**	T	T	T	T
12743	994	A	A	A	A	A	G	G	G
12839	1090	C	C	C	C	C	T	T	T
12842	1093	T	T	T	T	T	C	C	C
12872	1123	A	A	**G**	A	A	A	A	A
12875	1126	C	**T**	C	C	C	C	C	C
12881	1132	C	C	C	C	C	T	T	T
12899	1150	A	A	A	A	A	G	G	G
12926	1177	A	A	A	**G**	A	A	A	A
12962	1213	A	A	A	A	**G**	A	A	A
12974	1225	C	C	C	**A**	C	C	C	C
12992	1243	G	G	G	G	G	A	A	A
13031	1282	**C**	T	T	T	T	T	T	T
13034	1285	A	A	A	A	A	G	G	G
13062	1313	A	A	A	A	A	A	A	**G**
13064	1315	T	T	T	T	T	C	C	C
13073	1324	C	C	C	C	C	T	T	T
13079	1330	G	G	G	G	**A**	G	G	G

Position* relative to:		F clade					S clade		
NC_000934	Current dataset (4258 bp)	North-central (69)	East-central (50)	South-central (72)	West-central (27)	Western (1)	Savanna-wide (289)	Northern-savanna (47)	Southeast-savanna (98)
13082	1333	**C**	T	T	T	T	T	T	T
13104	1355	C	C	C	C	C	T	T	T
13112	1363	A	A	**C**	A	A	A	A	A
13134	1385	T	T	T	T	T	C	C	C
13181	1432	A	A	A	A	A	**G**	A	A
13186	1437	C	C	C	C	**T**	C	C	C
13187	1438	A	A	A	A	A	T	T	T
13190	1441	T	T	T	T	T	T	T	**C**
13208	1459	A	A	A	A	A	G	G	G
13250	1501	C	C	C	C	**T**	C	C	C
13262	1513	T	T	T	T	T	C	C	C
13280	1531	G	G	G	G	G	A	A	A
13309	1560	C	C	C	C	C	T	T	T
13319	1570	T	T	T	T	T	C	C	C
13330	1581	C	C	C	C	C	C	**T**	C
13333	1584	C	C	C	C	C	**T**	C	C
13352	1603	T	T	T	T	T	C	C	C
13358	1609	C	C	C	C	C	T	T	T
13409	1660	A	A	**G**	A	A	A	A	A
13424	1675	C	C	C	C	C	T	T	T
13427	1678	G	G	G	G	G	A	A	A
13439	1690	T	T	**C**	T	T	T	T	T
13450	1701	C	C	C	**T**	C	C	C	C
13472	1723	C	C	C	C	C	T	T	T
13492	1743	T	T	T	T	T	T	**C**	T
13589	1840	C	C	**T**	C	C	C	C	C
13616	1867	C	C	C	C	C	T	T	T
13701	1952	C	C	C	C	C	T	T	T
13704	1955	T	T	T	T	**C**	T	T	T
13750	2001	A	A	A	A	**G**	A	A	A
13762	2013	C	C	C	C	C	T	T	T
13775	2026	C	C	C	C	C	**T**	C	C
13780	2031	A	A	A	A	A	G	G	G
13801	2052	T	T	T	T	T	C	C	C
13823	2074	T	T	T	T	**C**	T	T	T
13857	2108	A	A	**G**	A	A	A	A	A
13859	2110	A	A	A	**G**	A	A	A	A
13874	2125	T	T	T	T	T	C	C	C
13889	2140	T	T	T	T	T	T	T	**C**
13919	2170	G	G	G	G	G	A	A	A
13936	2187	T	T	T	T	T	C	C	C
13946	2197	T	T	T	T	T	C	C	C
13955	2206	C	C	**T**	C	C	C	C	C
13958	2209	T	T	T	T	T	G	G	G
13967	2218	T	T	T	T	T	C	C	C
13997	2248	A	A	A	A	A	G	G	G
14024	2275	A	**G**	A	A	A	A	A	A
14038	2289	G	G	**A**	G	G	G	G	G
14101	2352	A	A	A	A	**G**	A	A	A
14140	2391	C	C	C	C	C	T	T	T
14141	2392	Y	Δ	T	T	T	C	C	C
14142	2393	A	Δ	A	A	A	A	A	A
14173	2424	T	T	T	T	T	C	C	C
14174	2425	C	C	C	C	C	T	T	T
14231	2482	G	G	G	G	G	A	A	A
14291	2542	C	C	C	C	C	T	T	T
14293	2544	A	A	A	**G**	A	A	A	A
14311	2562	T	T	T	T	**C**	T	T	T
14353	2604	C	C	C	C	C	T	T	T
14371	2622	C	C	C	C	**T**	C	C	C
14410	2661	T	T	T	T	T	C	C	C
14419	2670	C	C	C	C	C	**T**	C	C
14461	2712	A	A	A	A	A	G	G	G
14482	2733	C	C	C	C	C	T	T	T
14509	2760	A	A	A	A	G	A	A	A
14524	2775	C	C	C	C	**T**	C	C	C
14593	2844	C	C	C	C	C	T	T	T
14605	2856	T	T	T	T	T	C	C	C

Position* relative to:		F clade					S clade		
NC_000934	Current dataset (4258 bp)	North-central (69)	East-central (50)	South-central (72)	West-central (27)	Western (1)	Savanna-wide (289)	Northern-savanna (47)	Southeast-savanna (98)
14608	2859	C	C	C	C	C	T	T	T
14611	2862	C	C	C	C	C	T	T	T
14680	2931	C	C	C	C	C	T	T	T
14763	3014	A	A	A	A	**G**	A	A	A
14812	3063	G	G	G	G	G	G	G	**A**
14824	3075	C	C	C	C	C	T	T	T
14870	3121	T	T	T	T	T	C	C	C
14881	3132	A	A	A	A	A	**G**	A	A
14905	3156	T	T	T	T	**C**	T	T	T
14914	3165	T	T	T	T	T	C	C	C
14926	3177	T	T	T	T	T	C	C	C
14947	3198	C	C	C	C	C	T	T	T
14953	3204	A	A	A	A	A	G	G	G
14968	3219	C	C	C	C	C	T	T	T
15041	3292	C	C	C	**T**	C	C	C	C
15076	3327	C	**T**	C	C	C	C	C	C
15080	3331	T	T	T	T	T	C	C	C
15145	3396	T	T	T	T	T	A	A	A
15184	3435	T	T	T	T	**C**	T	T	T
15196	3447	C	C	C	C	C	T	T	T
15199	3450	C	C	C	C	C	T	T	T
15220	3471	T	**C**	T	T	T	T	T	T
15232	3483	T	T	T	T	T	C	C	C
15242	3493	T	T	T	T	T	C	C	C
15271	3522	C	C	C	C	T	C	C	C
15300	3551	C	C	C	C	C	T	T	T
15332	3583	A	A	A	A	A	G	G	G
15396	3647	A	**G**	A	A	A	A	A	A
15401	3652	T	T	T	T	**C**	T	T	T
15420	3671	A	A	A	A	A	A	A	**G**
15425	3676	A	A	A	A	A	Δ	Δ	Δ
15425	3677	C	C	C	C	C	T	T	T
15426	3678	C	C	C	**T**	C	C	C	C
15591	3843	T	**C**	T	T	T	T	T	T
15620	3872	T	T	T	T	T	**C**	T	T
15631	3883	T	T	T	T	**C**	T	T	T
15691	3943	C	C	C	C	**T**	C	C	C
15743	3995	C	C	C	C	C	C	**T**	C
15983	4235	C	C	C	C	C	T	T	T
15987	4239	T	T	T	T	T	A	A	A
No. of subclade specific diagnostic sites		4	8	10	15	24	8	5	6

**Two positions are indicated for nucleotides; the first position is relative to the *Loxodonta africana* complete mitochondrial genome (GenBank No: NC_000934); the second position is relative to our 4258 bp mtDNA alignment. Subclade specific nucleotide site character states are bold and lightly shaded. Numbers in parentheses indicate our sample sizes for elephants for each subclade. Delta indicates a deletion.

Table S4. Geographic distribution of mtDNA haplotypes (4258 bp).

Country	Locality	Haplotype found in						Total no. of elephants
		Single individual	One locality	Two localities	Three localities	Four localities	Eight localities	
Botswana	CH	0	0	1	13	19	12	45
Botswana	MA	0	6	3	0	12	1	22
Botswana	SA	4	2	5	1	14	1	27
Cameroon	BE	2	0	7	0	0	0	9
Cameroon	WA	2	12	12	0	0	0	26
CAR	DS	2	50	2	0	0	0	54
DRC	GR	8	12	0	0	0	0	20
Gabon	LO	1	9	7	0	0	0	17
Kenya	AB	0	4	2	1	9	1	17
Kenya	AM	3	33	23	9	2	0	70
Kenya	KE	3	9	38	9	8	0	67
Kenya	MK	0	0	0	0	3	0	3
Namibia	NA	2	50	0	0	0	8	60
Sierra Leone	SL	1	0	0	0	0	0	1
ROC	OD	1	0	2	0	0	0	3
S. Africa	KR	3	25	21	1	0	0	50
Tanzania	NG	1	0	6	5	0	3	15
Tanzania	SE	0	17	1	1	0	0	19
Tanzania	TA	2	11	2	10	0	21	46
Zambia	ZZ	1	0	4	0	10	2	17
Zimbabwe	HW	3	2	16	14	15	0	50
Zimbabwe	SW	2	2	7	1	3	0	15
Total no. of elephants		41	244	159	65	95	49	653
Percent of elephants		6.28	37.37	24.35	9.95	14.55	7.50	100.00

Results based on 4258 bp of mtDNA sequence from *MT-ND5* to control region. Listed for each category are the number of elephant individuals.

CAR: Central African Republic, DRC: Democratic Republic of the Congo, ROC: Republic of the Congo

Table S5. Geographic distribution of control region haplotypes.

Country	Haplotype found in							Total
	Single country	Two countries	Three countries	Four countries	Five countries	Six countries	Eight countries	
Angola	1	0	0	0	0	0	0	1
Botswana	2	2	3	1	1	1	1	11
Cameroon	7	5	4	1	0	0	0	17
CAR	4	4	3	1	0	0	0	12
Chad	0	0	1	0	0	0	0	1
Côte d'Ivoire	3	0	0	0	0	0	0	3
DRC	7	2	3	3	1	0	0	16
Gabon	9	4	3	0	0	0	0	16
Ghana	2	3	2	0	0	0	0	7
Kenya	6	5	2	2	0	1	1	17
Liberia	0	0	1	0	0	0	0	1
Mali	1	3	0	0	0	0	0	4
Mozambique	1	0	0	0	0	0	0	1
Namibia	5	0	1	0	1	0	1	8
ROC	1	3	2	1	0	0	0	7
Sierra Leone	0	0	1	0	0	0	0	1
S. Africa	2	1	1	1	0	1	1	7
Sudan	0	0	1	0	0	0	0	1
Tanzania	3	0	2	1	0	1	1	8
Uganda	1	3	2	2	0	0	1	9
Zambia	0	0	1	1	1	1	1	5
Zimbabwe	8	3	3	2	1	1	1	19
Total	63	19	12	4	1	1	1	101

Based on 316 bp of control region sequences from the current and previous transnational studies (Eggert et al. 2002; Nyakaana et al. 2002; Debruyne et al. 2003; Debruyne 2005; Johnson et al. 2007).
Listed for each category are the number of haplotypes.
"Haplotype" refers to a distinct mtDNA sequence, which may be carried by one or more elephants.
Column totals will be less than the sum of rows for haplotypes found in multiple countries.
CAR: Central African Republic, DRC: Democratic Republic of the Congo, ROC: Republic of the Congo

Table S6. Population pairwise F_{ST} and F_{ST} *P* values for mtDNA.

(A) Population pairwise F_{ST} of mtDNA

	AB	AM	BE	CH	DS	GR	HW	KE	KR	LCY	LO	MA	MK	NA	NG	OD	SA	SE	SW	TA	WA	ZZ
AB	0.00																					
AM	0.27	0.00																				
BE	0.70	0.87	0.00																			
CH	0.20	0.34	0.31	0.00																		
DS	0.95	0.96	0.51	0.69	0.00																	
GR	0.90	0.95	0.34	0.57	0.61	0.00																
HW	0.42	0.58	0.20	0.10	0.58	0.45	0.00															
KE	0.15	0.36	0.78	0.29	0.93	0.89	0.50	0.00														
KR	0.50	0.64	0.75	0.27	0.92	0.88	0.39	0.46	0.00													
LCY	0.95	0.98	0.03	0.49	0.90	0.74	0.36	0.92	0.90	0.00												
LO	0.93	0.96	0.38	0.62	0.87	0.76	0.53	0.91	0.90	0.75	0.00											
MA	0.72	0.82	0.72	0.28	0.95	0.90	0.33	0.61	0.09	0.94	0.92	0.00										
MK	-0.02	0.45	0.50	0.09	0.95	0.87	0.33	0.23	0.51	1.00	0.92	0.75	0.00									
NA	0.20	0.21	0.83	0.29	0.95	0.93	0.54	0.34	0.59	0.96	0.95	0.77	0.35	0.00								
NG	0.63	0.81	0.08	0.26	0.57	0.21	0.19	0.73	0.69	0.30	0.57	0.66	0.48	0.77	0.00							
OD	0.91	0.96	-0.12	0.47	0.49	0.38	0.32	0.90	0.88	0.25	0.54	0.90	0.83	0.94	0.14	0.00						
SA	0.87	0.93	0.40	0.51	0.72	0.55	0.34	0.88	0.87	0.72	0.74	0.87	0.85	0.91	0.40	0.49	0.00					
SE	0.92	0.95	0.38	0.58	0.68	0.09	0.46	0.90	0.89	0.80	0.80	0.91	0.90	0.94	0.20	0.48	0.59	0.00				
SW	0.46	0.67	0.63	0.17	0.93	0.86	0.30	0.41	0.04	0.89	0.89	0.12	0.43	0.59	0.57	0.84	0.84	0.88	0.00			
TA	0.07	0.13	0.60	0.12	0.86	0.79	0.37	0.20	0.38	0.79	0.82	0.47	0.00	0.08	0.53	0.76	0.78	0.80	0.28	0.00		
WA	0.40	0.62	0.05	0.15	0.58	0.43	0.14	0.49	0.49	0.22	0.45	0.44	0.28	0.58	0.14	0.18	0.45	0.44	0.36	0.36	0.00	
ZZ	0.53	0.74	0.13	0.12	0.65	0.43	0.05	0.65	0.62	0.29	0.56	0.58	0.37	0.70	0.12	0.23	0.24	0.45	0.48	0.45	0.13	0.00

(B) F_{ST} *P* values

Number of permutations: 10,100

	AB	AM	BE	CH	DS	GR	HW	KE	KR	LCY	LO	MA	MK	NA	NG	OD	SA	SE	SW	TA	WA	ZZ
AB	*																					
AM	0.000+-0.000	*																				
BE	0.001+-0.000	0.001+-0.000	*																			
CH	0.000+-0.000	0.000+-0.000	0.004+-0.001	*																		
DS	0.000+-0.000	0.000+-0.000	0.003+-0.001	0.000+-0.000	*																	
GR	0.000+-0.000	0.000+-0.000	0.006+-0.001	0.000+-0.000	0.000+-0.000	*																
HW	0.000+-0.000	0.000+-0.000	0.001+-0.000	0.000+-0.000	0.000+-0.000	0.000+-0.000	*															
KE	0.000+-0.000	0.000+-0.000	0.001+-0.000	0.000+-0.000	0.000+-0.000	0.000+-0.000	0.000+-0.000	*														
KR	0.166+-0.004	0.068+-0.002	0.600+-0.005	0.086+-0.003	0.999+-0.000	0.152+-0.004	0.088+-0.003	0.099+-0.003	*													
LCY	0.000+-0.000	0.000+-0.000	0.003+-0.001	0.000+-0.000	0.000+-0.000	0.000+-0.000	0.000+-0.000	0.000+-0.000	0.165+-0.004	*												
LO	0.000+-0.000	0.000+-0.000	0.001+-0.000	0.000+-0.000	0.000+-0.000	0.000+-0.000	0.000+-0.000	0.000+-0.000	0.089+-0.003	0.000+-0.000	*											
MA	0.001+-0.000	0.000+-0.000	0.006+-0.001	0.000+-0.000	0.000+-0.000	0.000+-0.000	0.000+-0.000	0.000+-0.000	0.242+-0.004	0.001+-0.000	0.001+-0.000	*										
MK	0.000+-0.000	0.000+-0.000	0.004+-0.001	0.000+-0.000	0.000+-0.000	0.000+-0.000	0.000+-0.000	0.000+-0.000	0.067+-0.002	0.000+-0.000	0.000+-0.000	0.000+-0.000	*									
NA	0.000+-0.000	0.000+-0.000	0.006+-0.001	0.000+-0.000	0.000+-0.000	0.000+-0.000	0.000+-0.000	0.000+-0.000	0.183+-0.004	0.000+-0.000	0.000+-0.000	0.004+-0.001	0.000+-0.000	*								
NG	0.047+-0.002	0.031+-0.002	0.350+-0.005	0.057+-0.003	0.067+-0.002	0.107+-0.003	0.049+-0.000	0.036+-0.002	0.999+-0.000	0.057+-0.002	0.044+-0.002	0.108+-0.003	0.075+-0.003	0.125+-0.003	*							
OD	0.000+-0.000	0.000+-0.000	0.001+-0.000	0.000+-0.000	0.000+-0.000	0.000+-0.000	0.000+-0.000	0.000+-0.000	0.285+-0.005	0.000+-0.000	0.000+-0.000	0.000+-0.000	0.000+-0.000	0.000+-0.000	0.047+-0.002	*						
SA	0.000+-0.000	0.000+-0.000	0.000+-0.000	0.000+-0.000	0.000+-0.000	0.000+-0.000	0.000+-0.000	0.000+-0.000	0.150+-0.004	0.000+-0.000	0.000+-0.000	0.000+-0.000	0.000+-0.000	0.000+-0.000	0.007+-0.001	0.000+-0.000	*					
SE	0.000+-0.000	0.000+-0.000	0.004+-0.000	0.000+-0.000	0.000+-0.000	0.000+-0.000	0.000+-0.000	0.000+-0.000	0.304+-0.005	0.000+-0.000	0.000+-0.000	0.003+-0.001	0.000+-0.000	0.000+-0.000	0.117+-0.003	0.000+-0.000	0.000+-0.000	*				
SW	0.000+-0.000	0.000+-0.000	0.001+-0.000	0.000+-0.000	0.000+-0.000	0.000+-0.000	0.000+-0.000	0.000+-0.000	0.110+-0.003	0.000+-0.000	0.000+-0.000	0.000+-0.000	0.000+-0.000	0.000+-0.000	0.031+-0.002	0.000+-0.000	0.000+-0.000	0.000+-0.000	*			
TA	0.000+-0.000	0.000+-0.000	0.005+-0.001	0.000+-0.000	0.000+-0.000	0.000+-0.000	0.000+-0.000	0.000+-0.000	0.112+-0.003	0.000+-0.000	0.000+-0.000	0.001+-0.000	0.000+-0.000	0.000+-0.000	0.074+-0.003	0.000+-0.000	0.000+-0.000	0.000+-0.000	0.000+-0.000	*		
WA	0.000+-0.000	0.000+-0.000	0.002+-0.000	0.000+-0.000	0.000+-0.000	0.000+-0.000	0.000+-0.000	0.000+-0.000	0.218+-0.005	0.000+-0.000	0.000+-0.000	0.002+-0.000	0.000+-0.000	0.000+-0.000	0.048+-0.002	0.000+-0.000	0.000+-0.000	0.000+-0.000	0.000+-0.000	0.000+-0.000	*	
ZZ	0.000+-0.000	0.000+-0.000	0.008+-0.001	0.000+-0.000	0.000+-0.000	0.000+-0.000	0.000+-0.000	0.000+-0.000	0.475+-0.005	0.000+-0.000	0.000+-0.000	0.002+-0.000	0.000+-0.000	0.000+-0.000	0.189+-0.004	0.000+-0.000	0.000+-0.000	0.000+-0.000	0.000+-0.000	0.000+-0.000	0.000+-0.000	*

Table S7. List of sample locations (shown as numbers in Figure 1).

No.	Country	Location	Sample origin
Nyakaana et al., 2002			
1	Ghana		Wild
2	Uganda	Murchison Falls NP	Wild
3	Uganda	Queen Elizabeth NP	Wild
4	Uganda	Kidepo Valley NP	Wild
5	Kenya	Samburu GR	Wild
6	Kenya	Masai Mara NR	Wild
7	Kenya	Amboseli NP	Wild
8[df]	Namibia	Khorixas	Wild
9[d]	Namibia	Caprivi NP	Wild
10[d]	Botswana	Chobe NP	Wild
11[d]	Botswana	Kwando	Wild
12[d]	Botswana	Nunga Valley	Wild
13[d]	Botswana	Sibuyu FR	Wild
14[d]	Zimbabwe		Wild
15[d]	South Africa	Kruger NP	Wild
16[de]	Botswana	Ngwasha	Wild
Eggert et al., 2002			
17	Côte d'Ivoire	Tai NP	Wild
18	Ghana	Bia NP	Wild
19	Ghana	Kakum NP	Wild
20	Ghana	Mole NP	Wild
21	Ghana	Red Volta Valley	Wild
22	Mali	Gourma Region	Wild
23	Cameroon	Banyang Mbo Wildlife Sanctuary	Wild
24	Cameroon	Dja FR	Wild
25	Cameroon	Benoue NP	Wild
26	Cameroon	Waza NP	Wild
27	South Africa	Addo Elephant NP	Wild
28	Kenya		Zoo
Debruyne et al., 2003, Debruyne, 2005			
29	Liberia		Museum
30[h]	Sierra Leone		Museum, Zoo
31	Côte d'Ivoire		Museum
32	Chad	Zakouma NP	Museum
33	Sudan (ex-southern Egypt)		Museum
34	Cameroon		Museum
35	Cameroon	Yambong	Museum
36	Gabon	Coast near Aloombé	Museum
37	Gabon	Lope National Park	Wild
38	RC		Museum
39	DRC	Mai-Ndome Lake	Museum
40	DRC	Bosobolo	Museum
41	CAR	Ubangi River	Museum

No.	Country	Location	Sample origin
42	DRC	Uele n' Dungu	Museum
43	DRC	Kamemba	Museum
44	DRC	Kanyatsi	Museum
45	Tanzania		Zoo
46	DRC	Panga Na Bodio	Museum
47	DRC	Katanga	Museum
48	DRC	Moma	Museum
49	Angola	Luiza (DRC frontier)	Museum
50	Zambia		Zoo
51	Mozambique		Zoo
52	Zimbabwe		Zoo
53	Botswana		Zoo
54[f]	Namibia		Zoo
55	South Africa		Zoo
56[e]	Uganda		Zoo
Johnson et al., 2007[g]			
57	CAR	Dzanga-Sangha NP	Wild
58	RC	Noubalé-Ndoki NP	Wild
59	Gabon	Monts de Cristal	Wild
60	Gabon	Iguéla NP	Wild
61	Gabon	Ivindo NP	Wild
62	Gabon	Lope NP	Wild
63	Gabon	Ipassa Reserve	Wild
64	Gabon	Loango/Mayumba NP	Wild
65	Gabon	Massif de Chaillu	Wild
66	Gabon	Minkébé NP	Wild
67	Gabon	Plateaux Batéké NP	Wild
68	Gabon	Conkouati-Douli NP	Wild

Table is based on Ishida et al. (2011)

Abbreviations:
CAR (Central African Republic)
DRC (Democratic Republic of the Congo)
FR (Forest Reserve)
NP (National Park)
NR (National Reserve)
RC (Republic of the Congo)

[a]Detailed location information not provided
[b]Zoo sample
[c]Museum sample
[d]Detailed locations follow Eggert et al. (2002)
[e]Plots are missing in Fig. 1 by Johnson et al. (2007)
[f]Locations 8 and 54 have colors reversed in Fig.1 by Johnson et al. (2007)
[g]Some locations were not clearly indicated in Table 1 of Johnson et al. (2007) and it was not possible to identify which haplotypes were present at these locations.
[h]Original GenBank data is from Barriel et al. (1999)
